# Comparing the effects of active or passive music therapy on negative symptoms and cognitive function in patients with chronic schizophrenia

**DOI:** 10.12669/pjms.42.2.13719

**Published:** 2026-02

**Authors:** Yuanyuan Ji, Shengli Zheng, Liying Liu, Haojie Wu, Jiayi Gao

**Affiliations:** 1 Yuanyuan Ji Department of Psychiatry, The Seventh People’s Hospital of Wenzhou City, Wenzhou, Zhejiang Province 325000, P.R. China; 2 Shengli Zheng Department of Psychiatry, The Seventh People’s Hospital of Wenzhou City, Wenzhou, Zhejiang Province 325000, P.R. China; 3 Liying Liu Department of Psychiatry, The Seventh People’s Hospital of Wenzhou City, Wenzhou, Zhejiang Province 325000, P.R. China; 4 Haojie Wu Department of Psychiatry, The Seventh People’s Hospital of Wenzhou City, Wenzhou, Zhejiang Province 325000, P.R. China; 5 Jiayi Gao Department of Psychiatry, The Seventh People’s Hospital of Wenzhou City, Wenzhou, Zhejiang Province 325000, P.R. China

**Keywords:** Active music therapy, Passive music therapy, Chronic schizophrenia, Negative symptoms, Cognitive function, Intrinsic motivation

## Abstract

**Objective:**

Music therapy (MT) is a non-pharmacological approach that has been used for the treatment of depression, anxiety, emotional distress, and mood disorders. This study aimed to compare the intervention effects of active MT (such as singing, playing, and music composition) and passive MT (such as listening) on negative symptoms and cognitive function in patients with chronic schizophrenia.

**Methodology:**

Clinical records of 120 chronic schizophrenia patients who received active/passive MT in the male ward of The Seventh Peoples Hospital of Wenzhou City, China, from April, 2024 to February, 2025 were retrospectively analyzed. The cohort included 60 cases of active MT and 60 cases of passive MT. The scoring results of the Positive and Negative Symptom Scale (PANSS), Scale for the Assessment of Negative Symptoms (SANS), Montreal Cognitive Assessment (MoCA), and Intrinsic Motivation Inventory for Schizophrenia Research (IMI-SR) were compared between the two groups.

**Results:**

After intervention, the scores of PANSS general subscale, PANSS negative subscale, PANSS total score, and the SANS score in the active MT group were lower than those in the passive MT group (*P*<0.05). Active MT was associated with significantly higher MoCA and IMI-SR scores compared to the passive MT (*P*<0.05).

**Conclusions:**

Compared with passive MT, active MT is more effective in improving negative symptoms, cognitive function, and intrinsic motivation in patients with chronic schizophrenia. Active MT can improve negative symptoms in patients with chronic schizophrenia. Active MT improves cognitive function in patients with chronic schizophrenia.- Compared with passive MT, active MT has higher benefits in treating patients with schizophrenia.

## INTRODUCTION

Negative symptoms—such as emotional withdrawal, reduced speech output, and attentional deficits—are recognized as core features of schizophrenia and persist in over half of all patients, particularly those with chronic or first-episode illness.^1^,^2^ These symptoms are strongly associated with poor functional outcomes and diminished quality of life.^2^,^3^ In parallel, cognitive impairments—including deficits in memory, attention, and executive functioning—are pervasive in schizophrenia and further hinder recovery.^3^-^6^ Unfortunately, conventional antipsychotic medications exert limited effects on both negative symptoms and cognitive deficits and are frequently accompanied by undesirable side effects.^1^-^4^

Music therapy (MT), a non-pharmacological and non-invasive intervention, has been widely applied in psychiatric care to modulate emotional regulation, promote social interaction, and stimulate neurophysiological activity.^7^,^8^ Previous studies have demonstrated its efficacy in reducing psychiatric symptoms across various conditions such as post-traumatic stress disorder, depression, and stroke.^7^-^9^ Moreover, MT has shown promise in improving negative symptoms and enhancing emotional expression among patients with schizophrenia, especially those who are treatment-resistant or unable to tolerate pharmacological regimens.^7^-^10^

Depending on the level of participation, music therapy can be broadly categorized into active MT—where individuals engage directly through singing, playing instruments, or composing music—and passive MT, which primarily involves listening to music.^11^ While MT has gained attention as an adjunctive treatment for psychiatric disorders,^8^-^10^,^12^ the literature remains limited in addressing whether active and passive modalities exert differential therapeutic effects. A small number of studies in dementia care suggest that combined or interactive forms may offer superior cognitive benefits,^12^ but direct comparisons in schizophrenia populations—particularly focused on negative symptoms and cognitive outcomes—are sparse.

This study aims to address this gap by directly comparing the effectiveness of active and passive MT interventions in patients with chronic schizophrenia. Through a structured analysis of their impact on negative symptoms, cognitive function, and intrinsic motivation, we seek to contribute to evidence-based, personalized non-pharmacological interventions for this population.

## METHODOLOGY

The study analyzed retrospectively selected records of 120 chronic schizophrenia patients who received active MT or passive MT in the male ward of the Seventh People’s Hospital of Wenzhou City, China, from April 2024 to February 2025.

### Ethical approval:

The ethics committee of the Seventh People’s Hospital of Wenzhou City approved this study, number: 2024-11, Date: April 1^st^ 2024.

### Inclusion criteria:

- Complies with ICD-10 (F20) diagnosis for schizophrenia;^1^
- PANSS negative subscale, with at least two items having a minimum value of ≥ 4: blunted affect (N1), emotional withdrawal (N2), poor rapport (N3), passive social withdrawal (N4), and lack of spontaneity and fluency in dialogue (N6);^13^
- The patient is in a clinically stable condition, defined as meeting all of the following criteria: (1) Clinical Global Impression–Severity (CGI-S) score ≤ 3 as assessed by an attending psychiatrist; (2) no episodes of acute agitation or aggressive behavior in the past two weeks; (3) ability to perform basic self-care and daily living activities; and (4) no major adjustments in antipsychotic medication dosage in the past four weeks.
- The condition has been treated with a fixed type of antipsychotic medication for more than three months, and evaluated by at least one associate chief physician or higher;
- Education level of primary school or above;
- Age range between 18 and 60 years old.

### Exclusion criteria:

- Diagnosed with schizophrenia less than two years ago;
- Patients who have been hospitalized for less than three months due to mental illness;
- PANSS positive subscale (P1-P7) > 28;
- Alcohol/drug abusers;
- Patients with a known history of major neurological disorders (traumatic brain injury, epilepsy, stroke, dementia, or other neurodegenerative conditions);
- Patients with any comorbid psychiatric disorders other than schizophrenia, including but not limited to bipolar disorder, obsessive-compulsive disorder, schizoaffective disorder, or major depressive disorder with psychotic features, as defined by ICD-10 diagnostic criteria;

### Secondary negative symptoms, defined as any of the following:

(1) Calgary Depression Scale for Schizophrenia (CDSS) score >7;
(2) UKU neurological side-effect score > 1 in any of the following items: dystonia (2.2), rigidity (2.3), hypokinesia/akinesia (2.5), tremor (2.6), or motor akathisia; or
(3) UKU sedative side-effect item (1.3) score > 1.13

The intervention period for both groups was 12 weeks, with sessions held three times a week, each lasting approximately 45-60 minutes, for a total of 36 sessions. MT sessions were implemented by two psychiatric nurses. To ensure standardization and consistency, all active MT sessions followed a predefined intervention protocol developed jointly by two certified psychiatric nurses in collaboration with a senior music therapist. The protocol included clearly defined objectives, activity flow, duration, and materials for each session. The intervention was structured into three progressive phases::

- Adaptation (Weeks 1–4),
- Skill acquisition (Weeks 5–8), and
- Integration (Weeks 9–12).

Each phase incorporated specific musical tasks (rhythmic clapping, instrument playing, group improvisation) with increasing levels of cognitive, motor, and social demands. All interventionists underwent pre-study training and participated in weekly supervision meetings throughout the intervention period to ensure fidelity. Any deviations from the protocol were documented and addressed through quality assurance measures. A detailed version of the intervention protocol, including structured session templates and example activities for each phase, is provided in Supplement-I.

**Supplement-I:** Standardized Protocol for Active Music Therapy Intervention

Phase	Component	Description
Adaptation (Weeks 1–4)	Objective	Familiarization with instruments and rhythm; build group cohesion
	Activities	Rhythmic clapping, percussion with simple instruments (e.g., sand hammers, triangles)
	Materials	Simple percussion instruments, metronome, familiar folk rhythm tracks
	Duration	~45–60 min per session
Skill Acquisition (Weeks 5–8)	Objective	Develop basic musical skills; encourage group collaboration
	Activities	Melodic instrument playing (e.g., Karimba, harmonica), rhythm imitation games, guided improvisation
	Materials	Hand-held melodic instruments, visual cue cards, collaborative rhythm boards
	Duration	~45–60 min per session
Integration (Weeks 9–12)	Objective	Apply musical skills in group context; reflect on experiences
	Activities	Mini-group performances, emotion-based improvisation, discussion of emotional experience after sessions
	Materials	Instruments of choice, music playback devices, feedback prompts
	Duration	~45–60 min per session

Active MT with “participation - interaction - creation” as the core. The specific content included:

- ***Weeks 1-4 (Adaptation Period):*** Collective hitting of simple instruments (such as sand hammers and triangular irons), clapping along with the rhythm (training synchronicity); Learning to sing simple choral pieces (such as fragments of “Jasmine Flower”, training social interaction).
- ***Weeks 5-8 (Skill Period):*** Playing melodic instruments (such as Karimba and harmonica) in groups and collaborating between each group to complete a short piece of music; Improvisational creation (such as creating 2-4 bar rhythms based on one’s own emotions to express inner feelings).
- ***Week 9-12 (Integration Period):*** Producing small-scale music programs according to the previously selected plan and performing them within the group; Reviewing the entire intervention process and answering questions such as “Do you feel pleasure during music activities?”.

Passive MT with “listening imagination relaxation” as the core. Specific content included:

- ***Weeks 1-4 (Guided Listening):*** playing pure music without lyrics (such as Mozart’s “K448” and natural sound effects blends); Guiding patients to pay attention to the melodic changes of ups and downs.
- ***Week 5-8 (Music Imagination):*** Playing narrative music (such as Vivaldi’s “Four Seasons, Spring”); Guiding patients to make associations based on music (such as “imagining themselves walking in the garden”) and share their imagination.
- ***Week 9-12 (Emotional Connection):*** Playing nostalgic music familiar to the patient (such as popular songs from the patient’s youth); Encouraging them to recall relevant scenes (such as “what comes to mind when you hear this song”) and promoting emotional expression.

### Assessment tools:

### Positive and Negative Symptoms Scale (PANSS:

Consisting of 30 items, divided into general subscale (16 items), positive subscale (7 items), and negative subscale (seven items), with a total score of 30-210 points. The higher score indicates more severe condition.

The Scale for the Assessment of Negative Symptoms (SANS), including five dimensions: emotional apathy, decreased willpower, social impulsivity, attention deficit, and interest in life, with a total of 24 items. The total score ranges from 0 to 120 points, with higher scores indicating the severity of negative symptoms.

Montreal Cognitive Assessment (MoCA) Scale, which measures cognitive function (attention, memory, calculation, language, abstract thinking, immediate memory, attention, etc.), with a total score of 0-30 points, with<26 points indicating cognitive impairment.

Intrinsic Motivation Inventory for Schizophrenia Research (IMI-SR). The score selects three dimensions of “interest/pleasure”, “autonomy”, and “effort level” (a total of 12 items), using a seven points scoring system (1=completely disagree, 7=completely agree), with higher scores indicating stronger intrinsic motivation. We used a previously translated and psychometrically validated Chinese version of the IMI-SR scale (C-IMI), adapted from the original Intrinsic Motivation Inventory. Prior validation studies among Chinese-speaking populations have shown good reliability and construct validity. In this study, we further examined the internal consistency of the C-IMI in our sample of patients with chronic schizophrenia. The Cronbach’s α for the total scale was 0.87, and the subscales (interest/pleasure, perceived choice, and effort) ranged from 0.81 to 0.89, indicating good internal reliability. The evaluation was conducted at two time points: one week prior to the intervention and one day following its completion.

The assessment was completed by two attending psychiatric physicians who were unaware of the grouping situation.

### Statistical analysis:

Descriptive statistics were used to report the variables, while Q-Q plots were used to evaluate normality. For normally distributed continuous variables, the mean and standard deviation (SD) were used; for non-normally distributed variables, the median and interquartile range (IQR) were used. The Student t-test compared continuous variables with a normal distribution between two groups, while the paired t-test compared within-group changes. The Mann-Whitney U test was used to evaluate variables with non-normal distributions between two groups. In contrast, the Wilcoxon signed-rank test was employed for intra-group comparisons before and after. Categorical variables are represented as frequency and percentage, and the chi-square test is used to determine intergroup differences. The statistical significance is set to *P*<0.05. The above analysis was conducted using SPSS version 26.0 (IBM Corp, Armonk, NY, USA). In addition, a post-hoc power analysis was conducted to assess whether the sample size was adequate for detecting a meaningful effect in the primary outcome (PANSS negative subscale). Based on prior studies reporting a medium effect size of approximately Cohen’s d = 0.6 for music therapy interventions in schizophrenia,^9^ we used G*Power 3.1 with α = 0.05 and power = 0.80. The analysis indicated that at least 45 participants per group would be required. Our final sample of 60 patients per group (N = 120) exceeds this threshold and thus ensures sufficient statistical power to detect clinically meaningful differences.

## RESULTS

**Fig.1:**
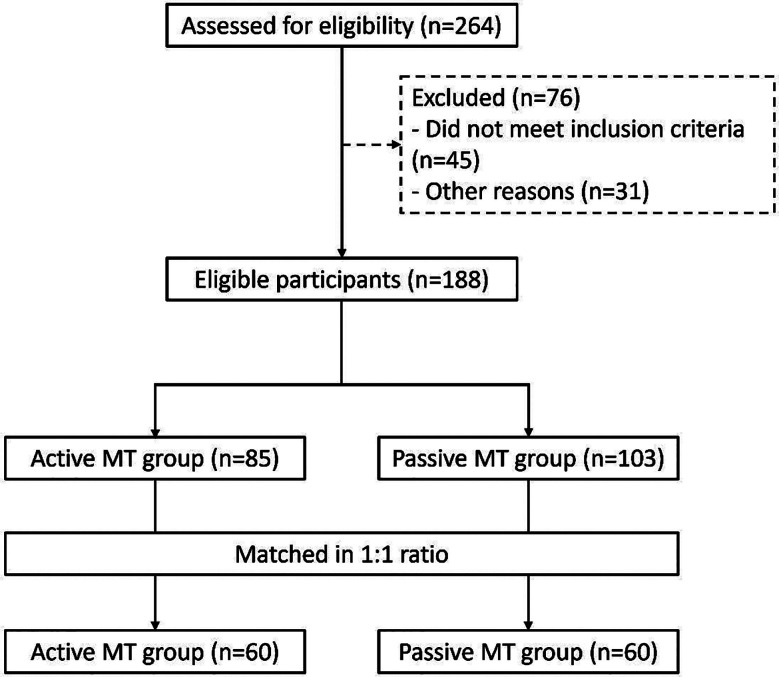
Patient screening process diagram. MT, music therapy.

This study included 120 patients with an age range of 18-60 years, and an average age of 40.2 ± 10.1 years. Among them, 60 patients who received active MT were matched with the queue of passive MT in a 1:1 ratio. The screening process of patients is shown in Fig.1. There was no statistically significant difference in general information, such as age, disease duration, education years, and baseline medication, between the two patient groups (P > 0.05) (Table-I).

**Table-I:** Comparison of clinical characteristics between two groups of patients.

Variables	Active MT group (n=60)	Passive MT group (n=60)	t/Z/χ^2^	P
Age (years), mean±SD	39.4±10.0	41.1±10.2	-0.932	0.353
BMI (kg/m^2^), mean±SD	24.6±2.9	23.9±3.0	1.221	0.225
Years of education, M(IQR)	9 (6.5-9)	9 (7-9)	-0.407	0.684
Family history (yes), n(%)	16 (26.7)	21 (35.0)	0.977	0.323
Disease duration (years), mean±SD	21.2±8.1	23.6±10.7	-1.349	0.18
Drug, n(%)			0.539	0.463
Single	35 (58.3)	31 (51.7)		
Merge	25 (41.7)	29 (48.3)		
Chlorpromazine equivalent dose (mg/d), mean±SD	272±85	262±70	0.732	0.466

***Note:*** MT, music therapy; BMI, body mass index; SD, standard deviation; M(IQR), median and interquartile range.

Before intervention, there was no significant difference in PANSS total score, as well as PANSS general, positive, and negative subscales, between the two groups (*P*>0.05). After intervention, the PANSS general subscale, PANSS negative subscale, PANSS total score, and PANSS positive subscale scores of the active MT group in both groups decreased compared to before intervention (P < 0.05). The active MT group had lower post-intervention PANSS general subscale, PANSS negative subscale, and total PANSS score compared to the passive MT group (P < 0.05) (Table-II).

**Table-II:** Comparison of PANSS scores between two groups before and after intervention

Time	Variables	Active MT group (n=60)	Passive MT group (n=60)	Z/t	P
Before intervention	PANSS general subscale, M(IQR)	33 (23-42.5)	33.5 (29-42.5)	-0.425	0.671
	Positive symptoms, M(IQR)	15 (13.5-17)	14 (12-16)	-1.798	0.072
	PANSS negative subscale, M(IQR)	23 (22-25)	25 (23-26.5)	-1.754	0.079
	PANSS total, M(IQR)	71 (61.5-80.5)	73 (66.5-79.5)	-0.431	0.667
After intervention	PANSS general subscale, M(IQR)	25 (22-28.5)^a^	33 (23-40.5)^a^	-3.481	0.001
	PANSS positive subscale, M(IQR)	14 (12-16)^a^	14 (12-16)	-0.309	0.757
	PANSS negative subscale, M(IQR)	15 (12-16)^a^	16.5 (14-20)^a^	-2.78	0.005
	PANSS total, mean±SD	54.8±6.3^a^	63.3±11.4^a^	-5.046	<0.001

***Note:*** Compared with before intervention in the same group,

a P<0.05. MT, music therapy; SD, standard deviation; M(IQR), median and interquartile range; PANSS, Positive and Negative Symptom Scale.

Before intervention, there was no significant difference in SANS, MoCA, and IMI-SR scores between the two groups (*P*>0.05). After intervention, the total scores of SANS in both groups decreased compared to before intervention, and was lower in the active MT group compared to the passive MT group (*P*<0.05). The MoCA and IMI-SR scores of both groups increased compared to before the intervention, and the active MT group had higher scores than the passive MT group (P < 0.05) (Table-III). In a descriptive analysis of the MoCA subdomains, the active MT group demonstrated more pronounced improvements in attention and delayed recall domains compared to the passive group. Specifically, the attention subscore increased from a baseline median of 4–5 points to 6–7 points after the intervention in the active MT group, whereas the passive MT group showed a more modest increase from 4–5 points to 5–6 points. Similarly, the delayed recall subscore improved from 2–3 points to 4–5 points in the active MT group, compared with an increase from 2–3 points to 3–4 points in the passive MT group. These domains are closely related to rhythm synchronization and memory engagement during active participation. Improvements in language and visuospatial/executive domains were present in both groups (typically increasing by 0–1 points), but the between-group differences were less distinct.

**Table-III:** Comparison of SANS, MoCA, and IMI-SR scores between two groups before and after intervention

Time	Variables	Active MT group (n=60)	Passive MT group (n=60)	t/Z	P
Before intervention	SANS, mean±SD	62.3±6.6	60.8±5.3	1.316	0.191
	MoCA, mean±SD	21.0±3.5	20.2±3.3	1.164	0.247
	IMI-SR, mean±SD	99.1±15.3	95.6±13.9	1.342	0.182
After intervention	SANS, mean±SD	47.7±6.5^a^	51.2±6.0^a^	-3.07	0.003
	MoCA, M(IQR)	27 (24-28)^a^	24 (22-27)^a^	-3.191	0.001
	IMI-SR, mean±SD	105.8±16.3^a^	100.0±13.5^a^	2.16	0.033

***Note:*** Compared with before intervention in the same group,

a P<0.05. MT, music therapy; SD, standard deviation; M(IQR), median and interquartile range; SANS, Scale for the Assessment of Negative Symptoms; MoCA, Montreal Cognitive Assessment; IMI-SR, Intrinsic Motivation Inventory for Schizophrenia Research.

## DISCUSSION

This study assessed the differences in the effects of active MT and passive MT interventions on PANSS, SANS, MoCA, and IMI-SR scores in patients with chronic schizophrenia. The results show that active MT reduced PANSS general subscale, PANSS negative subscale, PANSS total score, and SANS score, while significantly improving MoCA and IMI-SR scores.

The results of this study are consistent with the research results of Jiang et al.^14^ The main characteristic of patients with chronic schizophrenia is an imbalance in the activity of the autonomic nervous system, which is composed of the parasympathetic and sympathetic nervous systems.^15^ Heart rate variability (HRV) is a crucial indicator that reflects the balance of the autonomic nervous system. The parasympathetic nervous system primarily regulates high-frequency components (HF), while both the sympathetic and parasympathetic nervous systems jointly influence low-frequency components (LF).^15^,^16^ Thus, HF and the LF/HF serve as cardiac markers for parasympathetic and sympathetic ANS activation, respectively.^15^–^17^ McPherson et al.^18^ confirmed that rhythmic musical movement (active MT) reduces LF/HF, while passive MT increases LF/HF. This is consistent with the results of this study. The results showed that the PANSS negative subscale and SANS of the active MT group were significantly lower than those of the passive MT group. Such differences may be explained by the fact that activities such as active performance and choir allow patients to interact with structured music pulses to organize motor and neural activity.^14^,^18^ This type of movement may stimulate independent activation of the nervous and physiological systems, creating a new intervention experience that may regulate and target the physiological system, improving the PANSS negative subscale in patients with chronic schizophrenia.^19^–^21^

This study indicated that active MT is more effective in improving MoCA and IMI-SR scores compared to passive MT. Continuously playing along with the rhythm will keep the focus on hitting the beat accurately (avoiding mistakes), which can directly exercise one’s alertness and selective attention, and is beneficial for improving the score of the “attention” sub item in MoCA.^21^,^22^ Activating working memory, for example, by remembering which sound corresponds to each key on the musical instrument, is beneficial for neurogenesis in the hippocampus.^22^ Research has found that music training can increase the number of hippocampal neurons.^23^ Furthermore, active MT enhances the execution function: planning and arranging tracks will directly improve sub items such as “abstract thinking” and “delayed memory” in MoCA.^22^–^24^ Our domain-specific findings further support this interpretation. The most prominent improvements were observed in attention and delayed recall, which align with the cognitive demands of rhythm-following, beat matching, and short-term memory use during active musical engagement. These tasks may stimulate the prefrontal cortex and hippocampal circuits, reinforcing neural pathways associated with selective attention and memory encoding. In contrast, language and visuospatial improvements were more modest, possibly due to the less verbal or spatially complex nature of the intervention content. Therefore, active MT does have a certain effect on improving cognitive function in patients with chronic schizophrenia. Similarly, rhythmically playing with musical accompaniment (active MT) can satisfy people’s three basic psychological needs: “autonomy” (i.e. the ability to choose which instrument to play), “competence” (from “inability” to “ability to complete simple music”), and “sense of belonging” (recognition from the group), thereby generating intrinsic motivation.^11^,^18^,^25^ In addition, timely feedback can be obtained after playing (such as “this rhythm is very neat”), so playing is more helpful in improving self-efficacy and forming a virtuous cycle of “successful experience - more willing to participate - more success”. However, people who passively listen to music receive more indirect feedback from the outside world (such as “Do you think it sounds good?”), making it difficult for others to see their improvement.^18^,^25^,^26^ The results of this study also indicate that active MT intervention may be more ideal than passive MT intervention for improving cognitive abilities in patients with chronic schizophrenia.

While this study focused on quantitative outcomes using standardized psychometric instruments, it is important to recognize that active music therapy also involves inherently subjective experiences—such as emotional expression, creativity, and social connection—which may significantly influence therapeutic engagement. However, the present study did not include patient-reported outcomes such as emotional responses, satisfaction, or perceived therapeutic value. These experiential elements may mediate the clinical impact of active MT and should be explored in future studies through qualitative methods, such as patient interviews or observational assessments.

In addition to its clinical effects, the feasibility of implementing active music therapy (MT) in real-world settings warrants consideration.^18^ While hospital-based environments often have access to trained personnel and musical resources, community psychiatric clinics and rehabilitation centers may face logistical and staffing constraints.^27^ Nevertheless, recent developments in China’s community mental health services have introduced psychosocial rehabilitation programs—including music-related activities—led by social workers, psychiatric nurses, or peer-support volunteers.^14^ Simplified versions of active MT, such as group rhythmic clapping or structured sing-along sessions, may be adapted with minimal training and equipment.^25^ Future implementation studies should explore scalable, community-based models of active MT, including the use of telemedicine or mobile therapy units, to enhance accessibility and translational impact.

One possible explanation for the comparatively weaker effects observed in the passive music therapy group lies in the limited neural and psychological engagement associated with passive listening.^18^,^20^ Passive MT primarily activates auditory processing circuits and depends on affective resonance, both of which may be impaired in patients with chronic schizophrenia. Prior studies have shown that such patients often exhibit reduced auditory responsiveness and diminished emotional resonance, which may blunt the therapeutic impact of music-based auditory input.^1^,^13^,^21^ Moreover, passive MT involves minimal physical, cognitive, or interactive participation. This lack of multimodal stimulation may fail to activate the fronto-parietal networks responsible for executive functioning, attention control, and motivation regulation.^19^,^20^ In contrast, active MT provides goal-directed tasks that promote sensorimotor synchronization, voluntary expression, and interpersonal interaction, thereby engaging more extensive neural circuitry.^19^,^23^,^24^ This difference in neural engagement may contribute to the superior clinical outcomes observed in the active MT group.

The cross-cultural applicability of the intervention also warrants reflection. In this study, certain musical selections—such as traditional Chinese folk songs and nostalgic Mandarin-language tunes—were intentionally chosen to enhance emotional resonance and cultural familiarity among Mandarin-speaking patients.^21^ These selections may have triggered autobiographical memory and reinforced therapeutic engagement.^23^ However, we believe that the therapeutic framework of active music therapy—featuring rhythm-based interaction, group collaboration, and emotional expression—is not inherently culture-specific.^19^,^20^ Evidence from cross-cultural research in music therapy supports the generalizability of these core mechanisms across diverse linguistic and cultural groups.^28^ Therefore, with appropriate localization of musical content to align with cultural norms and preferences, this intervention model may be transferrable to non-Mandarin-speaking populations in both clinical and community settings.^24^

### Strength of the Study:

This is a rare and intuitive comparison of the differences in the effects of active and passive MT interventions on negative symptoms and cognitive function in patients with chronic schizophrenia. The improvement effect of a 12-week active MT session, based on participation, interaction, and creation, on negative symptoms, cognitive function, and intrinsic motivation in patients with chronic schizophrenia is significantly better than passive MT, which is based merely on listening and imagination.

### Limitations:

First, it was a single-center retrospective analysis conducted exclusively in a male psychiatric ward, which may limit the generalizability of the findings to other clinical settings or female populations. Second, the sample size was relatively small, and the use of retrospective data introduces risks of incomplete documentation, recall bias, and potential selection bias. Third, although therapists were uniformly trained, minor differences in facilitation style and patient engagement could have introduced variability in intervention fidelity. Fourth, we did not incorporate objective physiological or neuroimaging markers, such as heart rate variability (HRV), functional MRI, or dopamine levels. As such, the neurobiological mechanisms proposed in the discussion remain hypothetical and were not empirically validated. Fifth, while participants in both groups were matched on key demographic and clinical variables, we did not perform covariate-adjusted analyses to statistically control for confounders such as education level, illness duration, prior musical experience, or cultural familiarity with music. Sixth, the intervention protocol was relatively long (45–60 minutes per session), and some participants in the passive music therapy group may have experienced boredom or reduced motivation, potentially influencing the observed outcomes. Seventh, although we used a validated Chinese version of the IMI-SR scale, cultural differences in the interpretation of constructs like “autonomy” and “pleasure” may have influenced responses.

## CONCLUSION

This retrospective study suggests that active music therapy (MT) may be associated with greater improvements in negative symptoms, cognitive function, and intrinsic motivation compared to passive MT in patients with chronic schizophrenia. While these findings are encouraging, they should be interpreted with caution given the non-randomized design and modest sample size. In addition to its potential clinical utility, active MT appears to align with the values of modern psychiatric care—offering a non-pharmacological, low-risk, and patient-centered intervention. By promoting autonomy, emotional expression, and social engagement, active MT may serve as a supportive component of holistic rehabilitation programs. Future randomized controlled trials with physiological and neuroimaging-based endpoints are warranted to confirm these preliminary findings and clarify underlying mechanisms.

### Recommendations:

Future studies should consider applying cross-cultural validation techniques to ensure construct equivalence across populations. Finally, the study did not collect qualitative feedback or patient satisfaction data. As active music therapy inherently involves subjective experiences such as creativity, emotional expression, and social engagement, the absence of patient-centered feedback may have limited our ability to capture the full therapeutic value of the intervention. Future prospective studies are needed to address these limitations and explore mechanisms through more robust, multimodal approaches.

### Authors’ contributions:

**YJ:** Literature search, study design and manuscript writing.

**SZ, YL, HW and JG:** were involved in data collection, data analysis and interpretation. Critical review.

**YJ:** Manuscript revision and validation and is responsible for the integrity of the study. All authors have read and approved the final manuscript.
